# Open and Laparoscopic Colposuspension in Girls with Refractory Urinary Incontinence

**DOI:** 10.3389/fped.2017.00284

**Published:** 2017-12-22

**Authors:** Barbara Anna Dobrowolska-Glazar, Luitzen A. Groen, Anka J. Nieuwhof-Leppink, Aart J. Klijn, Tom P. V. M. de Jong, Rafal Chrzan

**Affiliations:** ^1^Department of Pediatric Urology, Jagiellonian University Medical College UCHC, Krakow, Poland; ^2^Department of Pediatric Urology, Academic Medical Center EKZ, Amsterdam, Netherlands; ^3^Department of Pediatric Urology, University Medical Center WKZ, Utrecht, Netherlands

**Keywords:** urinary incontinence, children, colposuspension, bladder neck insufficiency, laparoscopy

## Abstract

**Introduction:**

Lower urinary tract symptoms (LUTS) are very common in children. Standard treatments consist of urotherapy, antibiotic prophylaxis, anti-muscarinics, physical therapy, and the treatment of coexisting constipation. A small group of girls also present with stress incontinence or with stress-induced urge incontinence. In cases of persistent LUTS due to congenital bladder neck insufficiency (BNI), surgical treatment might be considered. The aim of this paper is to assess the results of open and laparoscopic colposuspension in children with refractory urinary incontinence (UI).

**Materials and methods:**

The results of 18 open and 18 laparoscopic consecutive colposuspensions were analyzed. All patients had UI and failed conservative treatment. BNI was proven by repeated perineal ultrasound and video-urodynamic study. The laparoscopic procedure was performed preperitoneally and the open procedure was *via* a transverse lower abdominal incision. The same postoperative protocol was used in both groups.

**Results:**

The mean operation time was 65 min for the open and 90 min for the lap procedure (*p* < 0.05). Full success was achieved in 7/18 in the open and in 8/18 in the lap group and partial response was seen in 3/18 and in 5/18, respectively (*p* = 0.64). No intraoperative complications occurred in this cohort.

**Conclusion:**

Open and laparoscopic colposuspension can be used to treat refractory UI in children with BNI when non-invasive methods fail.

## Introduction

Lower urinary tract symptoms are very common in children. Urinary incontinence (UI) is one of the most bothersome signs of lower urinary tract dysfunction. The majority of patients can be cured by means of standard urotherapy, and bowel management is often needed. Some patients require specific urotherapy (physical therapy, neuromodulation) and pharmacotherapy (1, 2). A small number of girls have persistent UI and bladder neck insufficiency (BNI). For those rare cases of congenital stress-incontinence or stress-induced detrusor overactivity, surgical treatment can be an option (3, 4).

The goal of the surgical procedure is to restore the anatomical relationships inside the pelvis. In this way, improvement of the function of the lower urinary tract can be achieved (5). The colposuspension procedure introduced by Burch has long been recognized as one of the most effective methods to cure stress urinary incontinence (SUI). The principle of this procedure is based on elevation of the bladder neck region by fixation of the anterior vaginal wall, to the left and right of the bladder neck, to Cooper’s ligament. Although mid-urethral slings are the first choice nowadays in adult uro-gynecology, colposuspension still remains a good alternative for those by whom slings are contraindicated and also in those who still wish to bear a child in their future (5–7). In adults, the outcomes of laparoscopic and open colposuspension are comparable (8).

The aim of this paper is to assess the results of open and laparoscopic colposuspension in children with refractory UI.

## Materials and Methods

**Figure 1:**
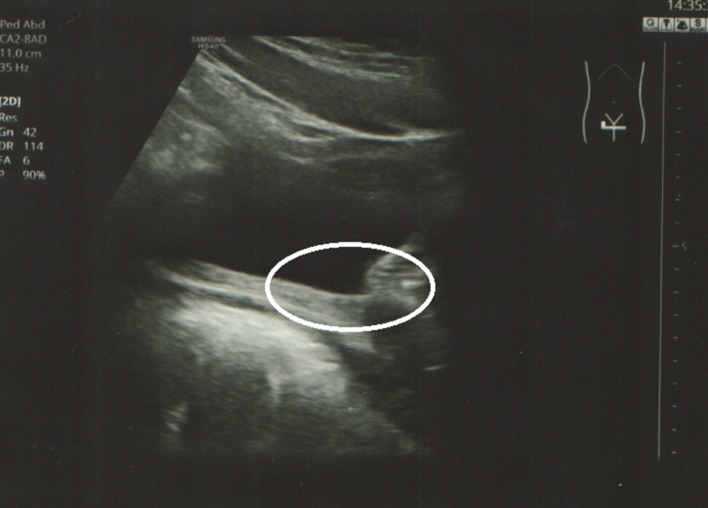
Transabdominal ultrasound: open bladder neck, sagittal view (circle).

**Figure 2:**
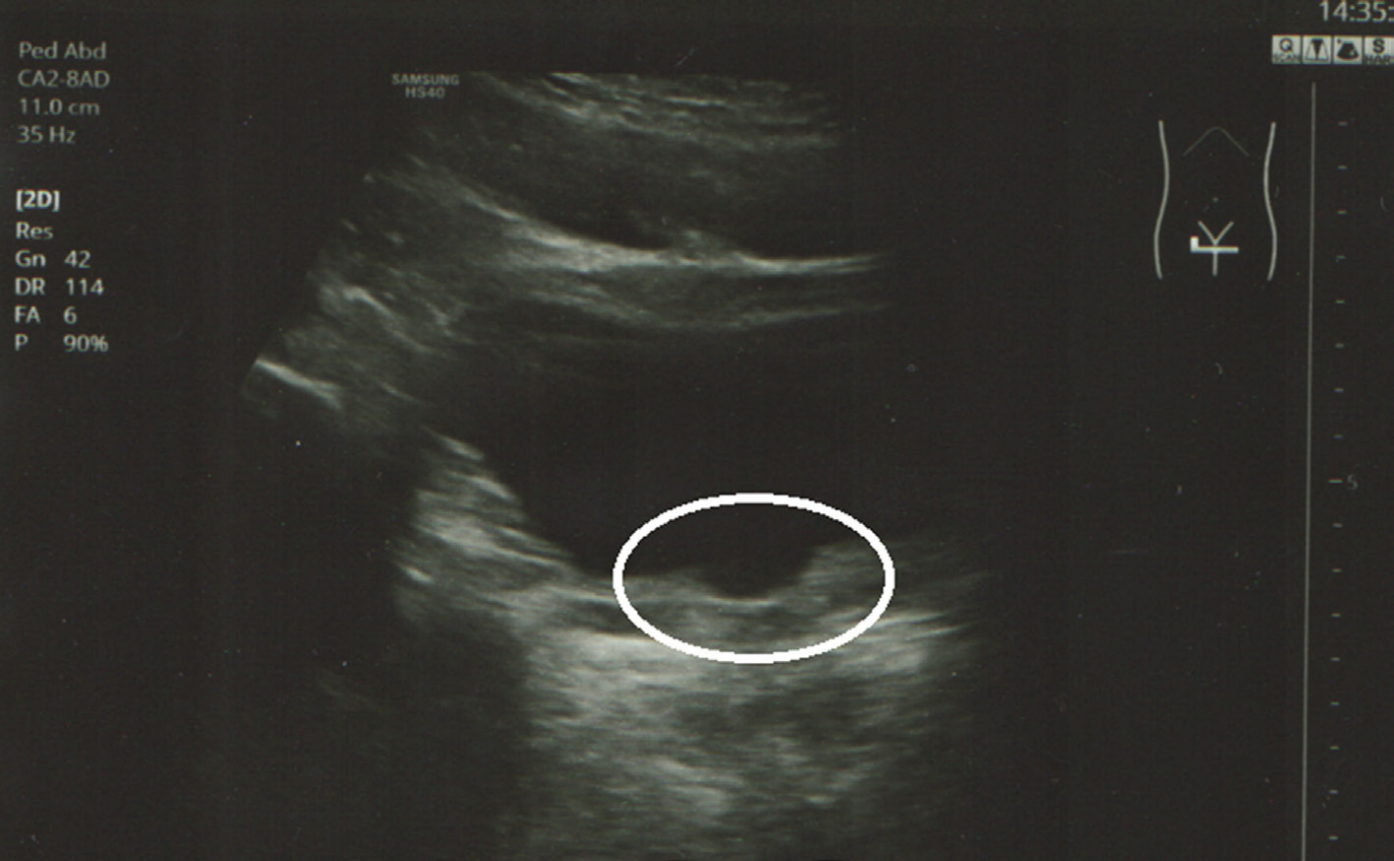
Transabdominal ultrasound: open bladder neck, horizontal view (circle).

**Figure 3:**
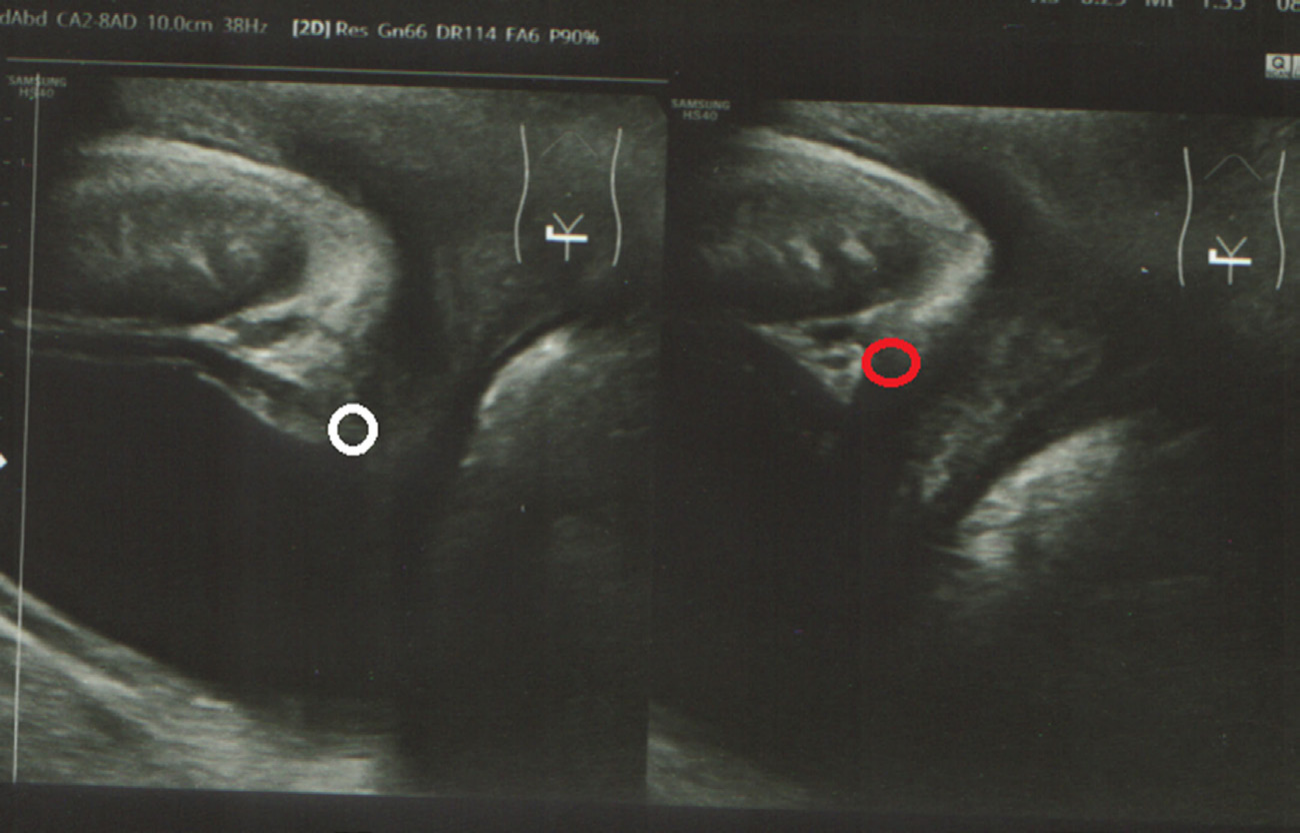
Perineal ultrasound: bladder neck hypermobility (circles).

**Figure 4:**
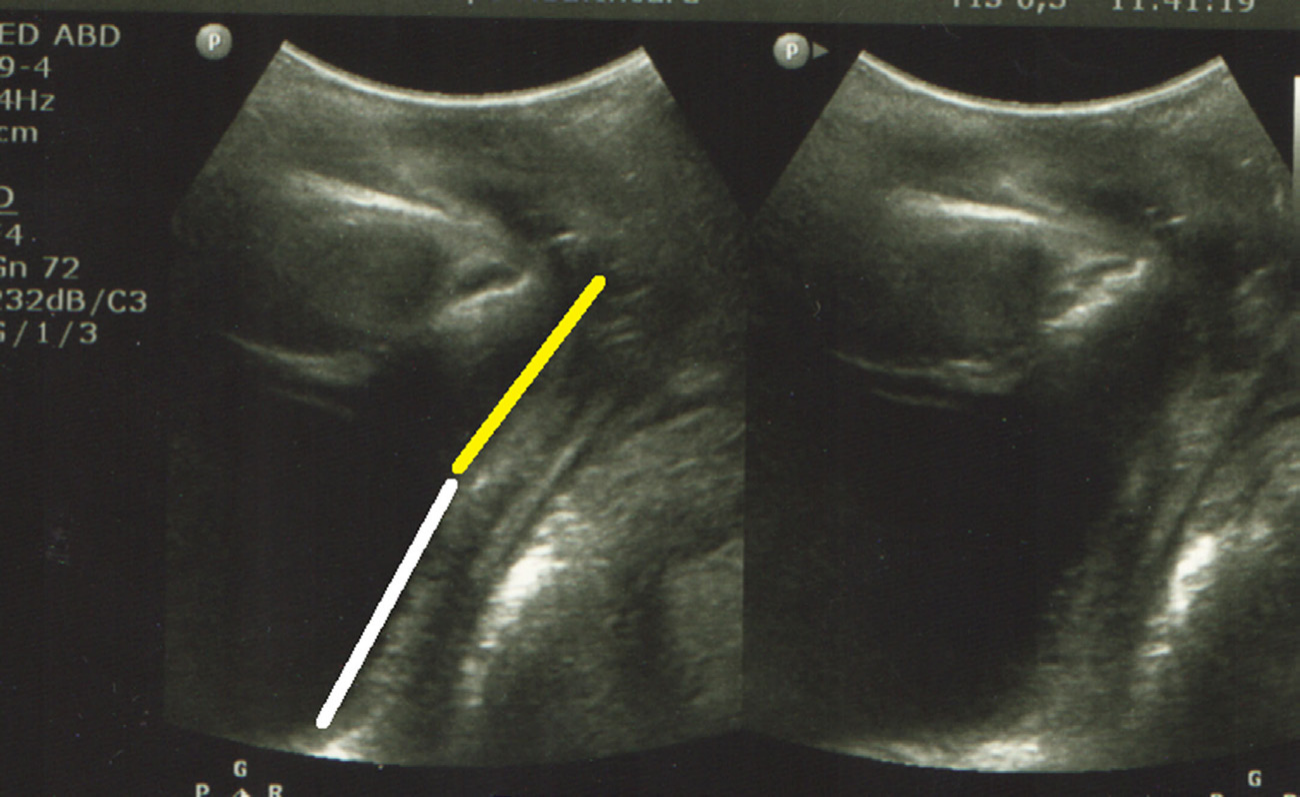
Perineal ultrasound: flat vesical urethral angle (parallel lines), Yellow line—posterior wall of the urethra and white line–posterior wall of the bladder.

Eighteen consecutive laparoscopic (LCS) and 18 consecutive open colposuspension (OCS) procedures were retrospectively analyzed. The only inclusion criterion was refractory UI based on BNI. Standardized diagnostic work-up consisting of a medical history, bladder and bowel diary, and physical examination with neurological assessment and uroflowmetry with assessment of the residual urine was done. A repeated transabdominal and perineal ultrasound (US) with the bladder neck appraisal (position and mobility in rest and during straining) as well as a video-urodynamic study (V-UDS) was were done in all patients. Bladder neck was considered insufficient based on the combination of the following features: an open bladder neck during filling phase (Figures 1 and 2), a hypermobile bladder neck region with a significant descent during straining (Figure 3), a flat vesicourethral angle (VUA) (Figure 4). The clinical symptoms before surgery are summarized in Table 1.

**Table 1:** Preoperative findings.

	Urinary incontinence	Constipation	Urge	UTIs
OCS	18	8	6	8
LCS	18	6	8	6

*OCS, open colposuspension; LCS, laparoscopic colposuspension; UTIs, urinary tract infections*.

All patients failed urotherapy in the ambulatory setting. All had an additional in-patient 10 day-in-hospital cognitive training program, which is the most intensive form of urotherapy for therapy-resistant cases, which also failed. All had been consulted by an experienced physical therapist with anal balloon biofeedback training of their pelvic floor. In the end, all patients suffered from refractory incontinence after failed conservative options for 2 years. Fourteen patients had urge and urge incontinence and did not respond to anti-muscarinics. Only three girls had leakage during V-UDS: two in the LCS group and one in the OCS group and this due to detrusor overactivity provoked by the open bladder neck (stress-induced overactivity). Bladder capacity was above 75% of the expected bladder capacity (EBC) and compliance was normal in all girls. Repeated uroflowmetry did not show any constant pattern in this group (bell-shaped, tower-shaped, and staccato-shaped curves were seen).

All patients were reviewed at 12 months after surgery. The outcomes were assessed as full response if the patient was completely dry, partial response in those who had fewer episodes of UI and failed if the degree of UI remained the same.

All patients were treated by Burch-type colposuspension. The mean age of the laparoscopically treated patients was 13.5 years and that of the patients treated by open surgery was 11.5 years. A urethrocystoscopy was done to evaluate the anatomy of the lower urinary tract in all children in the same session during colposuspension. A flat VUA was found in all patients before and good restoration (angulation) of the region was seen in all children after surgery.

The laparoscopic procedure was performed preperitoneally by means of three 5-mm ports, and the open procedure was *via* a transverse lower abdominal incision. The anterior wall of the vagina, lateral to the bladder neck, was mobilized and bilaterally sutured to Cooper’s ligament (polyglactin 2-0). The same postoperative protocol was used in both groups: the transurethral bladder catheter was removed on the fifth day and they were told to avoid physical exercise for 6 weeks. Balloon catheter 10 Fr was used in girls <10 years of age and 12 Fr in those ≥10 years of age. The Fisher’s exact test was used for the statistical analysis.

## Results

The mean operation time was 65 min for the open and 90 min for the laparoscopic procedure (*p* < 005). The overall success rate was 64%: full response in 15 (41.7%) and partial response in 8 (22.2%). Complete response (dryness) was achieved in 7/18 (39%) in the OCS and 8/18 (44%) in the LCS group and partial response was seen in 3/18 (17%) and 5/18 (28%), respectively (*p* = 0.64). Overall, 9 of the 14 (66%) girls were free of urinary tract infections (UTIs) and antibiotic prophylaxis could be ceased. Intraoperative complications were rare: one case of intraperitoneal CO_2_ leakage during laparoscopic procedure without the need for conversion. Postoperatively, two patients in the OCS and one in the LCS group needed temporary clean intermittent catheterization (CIC) due to urinary retention.

In all cases, perineal US was performed to ascertain that the operative procedure had resulted in a fixed urethra and bladder neck. In all cases, including those with a failed result, the bladder neck and urethra were adequately fixed and the congenital cystocele, when present preoperatively, was absent after surgery. No cases failed due to failed fixation of the bladder neck.

## Discussion

An insufficient bladder neck that remains continuously open during the filling phase of the bladder can provoke detrusor contractions. During straining, an insufficient bladder neck is very mobile and gets displaced deep in the pelvis. In our opinion, girls with refractory UI should undergo an extended diagnostic pathway, including evaluation of the bladder neck anatomy and function. There are no strict criteria for BNI. This diagnosis can be made by exclusion and by means of ultrasonography and V-UDS (2). The role of invasive urodynamics (UDS) in this group needs to be defined. It is always done in children with refractory incontinence to provide more information about the function of the lower urinary tract. However, one must keep in mind that the clinical symptoms are not always reproducible during cytometry and pressure-flow study, especially in children. In our small cohort only 3/36 had UI during V-UDS. Fluoroscopy during UDS can give additional clue on the anatomy of the bladder neck region but the same finding (an open bladder neck and its hypermobility as well as a flat VUA) can be assessed by means of the perineal US. That is why no standard UDS was done during the 1-year follow-up.

A small series showed that 50–75% of girls with refractory UI and insufficient bladder neck can be cured by a colposuspension procedure (3, 9, 10).

Due to its high cure rates, open colposuspension was the gold standard procedure for the treatment of UI in adult females for approximately 40 years. This procedure remains an excellent choice for the treatment of SUI in patients in whom the use of vaginal mesh is contraindicated. In the adult population, a good outcome can be reached in 85–90% of case (4). Colposuspension (open or laparoscopic) should be offered to women if a mid-urethral sling cannot be considered, which is a level A EAU Guidelines recommendation, among those patients who wish to get pregnant in the future (8). One should keep in mind that Burch procedures also have the lowest incidence of repeat SUI surgery in adult women (11). Taking this into consideration, Burch colposuspension seems to be an option in girls who require this type of surgery. Of course, colposuspension is not free from complications. In the adult population, detrusor overactivity and urinary retention are the most common side effects of this procedure (12–14). Since there is hardly any literature about the colposuspension in the pediatric patients, we can only hypothetically presume that the same complications can also occur in children but a longer follow-up and a bigger cohort are required for further evaluation. In our small cohort only three patients suffer from a transient urinary retention and needed CIC for a short period of time. This problem disappeared within a few weeks in all cases.

The laparoscopic approach combines the high efficacy of the Burch procedure with the minimal invasiveness of this technique. Although laparoscopy usually requires longer operating times, this procedure is related to a shorter hospital stay and better cosmetic outcome (5). Moreover, the laparoscopic colposuspension enables superior visualization the enlargement of the operating field and good hemostasis (15). Open colposuspension, especially in overweight patients, can be tedious, and bleeding from the venous convolutes in the anterior vaginal wall may cause problems. This may have been one of the reasons why adult urologists and gynecologists shifted to tapes. However, laparoscopy overcomes/prevents these problems. These observations have also been found in our series, but our cohort is too small to reach statistical significance. The role of laparoscopic colposuspension is questioned in adults because of the availability of mid-urethral slings. In the series by Jelovsek et al. and Ustün et al., transvaginal tape showed similar long-term efficacy as laparoscopic Burch for the treatment of SUI (16, 17). Nevertheless, the laparoscopic colposuspension is a dependable method for the treatment of SUI.

In the adult population, laparoscopic colposuspension has the same efficacy as the open procedure, showing a similar risk of voiding difficulty or *de novo* urgency. Carey et al. randomized 200 women to open or laparoscopic colposuspension, and Kitchener et al. reported a randomized controlled trial including 291 women. Both studies demonstrated no significant differences between laparoscopic and open colposuspension in objective and subjective measures at 24 months (18, 19). LCS was shown to be safe and feasible with a high success rate in the study by Köktürk et al. (6). Our small series shows that the observations of LCS made in the adult population are reproducible in children. Jenkins and Liu found that LCS is equivalent to open Burch colposuspension and there was no difference in subjective cure rates in comparison with tension-free slings too. They suggested that LCS is the procedure of choice in young women, because it avoids the potential complications of mesh (20). In children, a relatively smaller working space in the pelvis could be a technical issue in less experienced hands.

To the best of our knowledge, there is sparse literature on the use of the colposuspension procedure to treat refractory incontinence in otherwise healthy children. This means that no reference can be used to compare our results. In our study, 72% of girls were cured or improved after laparoscopic surgery and 56% of patients after the open procedure. These laparoscopic data are comparable to the 71% cure rate in adults presented by Carey et al. and Köktürk et al. (6, 18). In the study performed by Schmidt et al., the overall cure rate for the LCS was 82.6%, but in the first 40 patients it was only 69% (21), which means that the learning curve has an impact on the final results. As the pediatric population with SUI is incomparably smaller than the adult group, gaining the necessary experience is a much slower process. Open colposuspension gives an overall continence rate of between 85 and 90% in a Cochrane review presented by Lapitan et al., but it was only 56% in our study, as mentioned above (4). We do not know how to explain these differences, but we are convinced that the indications for this type of surgery in children must be clarified.

Injection of the bulking agents is another minimal invasive procedure that is used to cure SUI in the adults. In the pediatric population, it is only done in children with neurogenic lower urinary tract dysfunction and with complex congenital anomalies (exstrophy–epispadias complex) (22–24). To our knowledge, it is used on the other indications. This maneuver can increase infravesical resistance but it will not restore the anatomical relationships within the pelvis.

### Limitation of This Study

One limitation is the small number of patients. Furthermore, the objective criteria for BNI still do not exist. However, very careful and detailed additional work was performed by experienced pediatric urologists.

### Future Perspectives

There is certainly a need for prospective multicenter observational studies, as the group of girls with persistent incontinence is not large. Objective criteria to decide which patients will benefit most from colposuspension should be provided in the future. These criteria could be established based on patient characteristics, clinical symptoms, and additional tests e.g., UI, symptoms of bladder overactivity, bladder capacity, recurrent UTIs, and objective data from perineal ultrasonography/video-urodynamics (10).

## Conclusion

Open and laparoscopic colposuspension can be used to treat refractory UI in children with BNI when non-invasive methods fail; both are safe and effective. Strict inclusion criteria should be developed for girls with UI that can help to identify those who can benefit most from the surgical treatment.

According to the local rules and guidelines the Retrospective patient file research does not require approval from the Ethical Committee. Such research is only subject to the Agreement on Medical Treatment Act that has been signed by every patient and parents before any treatment was started.

## Ethics Statement

For this retrospective study no approval from the ethical committee was required.

## Author Contributions

BD-G, LG, AN-L, AK, TJ, and RC—preparation of the manuscript, contributions to the conception of the work, analysis and interpretation of data for the work, drafting the work and revising it critically for important intellectual content, and final approval of the version to be published, ensuring the integrity of any part of the work.

## Conflict of Interest Statement

The authors declare that the research was conducted in the absence of any commercial or financial relationships that could be construed as a potential conflict of interest.
